# Balancing Efficiency and Engagement: AI-Assisted Content for Research Communications in the RECOVER Initiative

**DOI:** 10.21203/rs.3.rs-7660686/v1

**Published:** 2025-09-26

**Authors:** Zoe Lewczak, Praveen Mudumbi, Janelle Linton, Maika Mitchell, Jasmine Briscoe, Pricilla Short, Nita Jain, Anisha Sekar, Alicia Chung

**Affiliations:** Harvard Medical School; New York University Grossman School of Medicine; NYU Grossman School of Medicine; NYU Grossman School of Medicine; NYU Grossman School of Medicine; NYU Grossman School of Medicine; Timeless Biosciences; RECOVER Patient, Caregiver, or Caregiver Representative; NYU Grossman School of Medicine

**Keywords:** Artificial Intelligence, Plain Language Summaries, Lay Language Research, Health Communications, Science Communications, Community Engagement, Media Promotion, Research Promotion

## Abstract

**Introduction:**

The growing availability of AI tools is transforming health and science communication by streamlining content creation and promotion. This study investigates the impact of AI-assisted research summaries on user engagement with the NIH-funded RECOVER program’s website and evaluates the efficiency and readability of the content.

**Methods:**

We analyzed Google Analytics 4 data from two distinct periods: one with entirely human-generated content and a second with AI-assisted content. We measured changes in page views, active users, and average engagement time, and assessed the review time and readability of the AI-enhanced summaries.

**Results:**

There was no significant change in page views or active users between the two periods. However, average engagement time increased by 4.37 seconds (P = .0461), suggesting AI-assisted content may be more compelling. Human review of AI-drafts averaged 19.88 changes, and readability improved, with the mean Flesch-Kincaid grade level decreasing from 12.28 to 11.56.

**Conclusion:**

This study demonstrates that AI can be a valuable tool for accelerating the creation of accessible and engaging content. Our findings highlight a crucial balance: while AI can save effort and reduce cost in public engagement efforts, human oversight remains essential to ensure the accuracy, clarity, and accessibility of vital health communications.

## Introduction

Communication is a cornerstone of any successful research initiative, and for health and science organizations, it is critical for establishing credibility and engaging the public. Effective scientific communication goes beyond simply reporting findings; it involves making complex information accessible, balanced, and respectful to a diverse audience.^1^ Technical manuscripts are like a professional chef’s intricate recipe—full of jargon and complex techniques that are inaccessible to most. Effective communication is the skilled cookbook author who translates that recipe into simple, step-by-step instructions so anyone can prepare and enjoy the result. The rapid advancement of Artificial Intelligence (AI) has revolutionized this field, offering new ways to streamline and enhance communication and media promotion efforts.^2^

A recent survey by the Institute for Public Relations found that 45% of organizations now use AI for various tasks, including content creation, ideation, and market research.^3^ For communication professionals, AI has become a powerful tool to synthesize vast amounts of information, identify key themes, and inform strategic messaging.^4^ This paper explores how the Researching COVID to Enhance Recovery (RECOVER) program, funded by the National Institutes of Health (NIH), has strategically integrated AI into its communication workflows. We focus on its use in drafting plain-language summaries and video scripts, which are essential for broad media promotion and public engagement.

## RECOVER Communications Teams using AI

NYU’s Clinical Science Core (CSC) for NIH RECOVER serves as the scientific leadership and operational backbone of the RECOVER Initiative, set up to better understand post-acute sequelae of SARS-CoV-2 infection, known as Long COVID. CSC has led all aspects of developing and conducting studies, including the dissemination of results for the RECOVER cohort studies at > 200 enrolling sites located in all 50 states and Puerto Rico. Since their inception in 2021, the RECOVER cohort studies enrolled a diverse population of nearly 30,000 adult and pediatric participants, collected > 50,000 biospecimens, and have over 140 reports published or under review.

To ensure this research reaches the widest possible audience, the CSC employs three distinct communication teams: Community Engagement, External Affairs, and internal Communications. These teams work in tandem to transform complex scientific findings into accessible formats for various media channels. A core principle of RECOVER is to ensure patient and community perspectives are integrated throughout the research and reflected in all communications.

Historically, scientific publications are written by and for the scientific community, making them difficult for non-specialists to understand. This creates a significant barrier to the public accessing potentially life-changing research.^5^ To bridge this gap, RECOVER’s communication teams have embraced Plain Language Summaries (PLS) and videos as crucial tools. A PLS translates complex research into easy-to-understand language, a process that is now being optimized with AI.^6^

**Figure 1:**
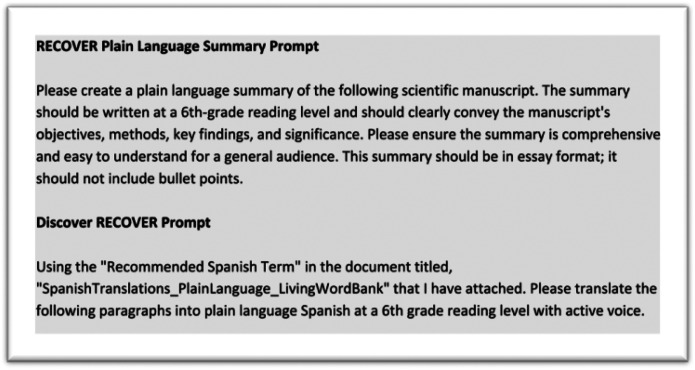
Sample prompts for PLS and Discover RECOVER video script generation

The team leverages **NYUChatGPT**, an AI tool developed by New York University (NYU) that leverages OpenAI’s GPT-3 technology to assist with a variety of tasks, including generating text-based content to help the NYU community.^7^ A finalized, peer-reviewed manuscript serves as the input for NYUChatGPT and is guided by a prompt. This carefully crafted prompt, aligned with our established plain language standards guide, is provided to NYUChatGPT (See Fig. 1). As a result, a plain language summary of the manuscript is returned. This use of AI allows the team to accelerate the creation of accessible content, thereby speeding up media promotion efforts and the overall dissemination of critical health information.

This prompt instructs NYUChatGPT to generate a PLS that adheres to specific readability levels (6th grade), avoids jargon, and effectively communicates the key findings and implications of the research to a non-scientific audience. This NYUChatGPT-produced initial draft serves as a foundation for the team to further refine and polish, ensuring accuracy, clarity, and accessibility for the target audience. The final draft is sent to the manuscript’s authors to ensure accuracy prior to publishing on the RECOVER website.

Digital media allows for user-friendly presentations of research findings: it puts evidence into practice, allowing health professionals and the public to better understand key research findings.^8^ Images and video can also be integral aspects of data collection, analysis, and reporting research studies.^9^ Digital media content has become a powerful platform for public engagement with science. By offering PLS in video format and incorporating infographics, we can more widely disseminate our findings. The Discover RECOVER series, launched by the Community Engagement (CE) team in collaboration with researchers and Patient, Caregiver, and Community Representatives, communicates manuscript findings in brief videos. The CE team utilizes NYUChatGPT in creating plain-language scripts for Discover RECOVER. AI is prompted to synthesize and summarize the key points of an uploaded manuscript in simple language. The CE team used the key points to draft the script in English. The script is then reviewed by the authors and the Patient, Caregiver, and Community Representatives, ensuring that the AI-generated content accurately reflects the key findings of the manuscript.

In alignment with RECOVER’s commitment to equal access in research, Discover RECOVER video scripts and infographics are translated into Spanish for improved access to findings. The CE team utilizes NYUChatGPT to draft the first version of Spanish subtitles and translated infographics for the videos. The CE team inputs the final English version of the Discover RECOVER script into NYUChatGPT and instructs the AI to generate a plain language script at a 6th grade reading level with active voice utilizing a Spanish Translations Plain Language Living Word Bank. This Spanish Translation Word Bank is a project managed by the CSC and Administrative Coordinating Center (ACC) Communications team that includes standard translation preferences for RECOVER deliverables. The AI-generated script is then shared with CSC Communications for quality assurance. The Communications team reviews the script to ensure the translation is in accordance with the preferred terms for RECOVER. This is an important step in the process as generative AI often makes translation errors, being unable to capture the complexity of healthcare information.

## Methods

To evaluate the effect of generative-AI enhanced content on web traffic and engagement for the RECOVER website, recovercovid.org, we used Google Analytics 4 (GA4) to compare user interactions from two distinct time periods: December 1, 2023 to September 10, 2024, when all content was human-generated, and September 10, 2024 to January 20, 2025, when NYUChatGPT was deployed to assist with all research summaries, written and video, in English and Spanish. GA4 was configured to collect data on the following metrics:

1. Views. The number of mobile app screens or web pages that users saw. Repeated views of a single screen or page are counted.
2. Active Users. The number of distinct users who visited the recovercovid.org website.
3. Average engagement time per active user. The average time that recovercovid.org was in focus in an active user’s browser or app, expressed in seconds.

We examined traffic and engagement metrics on:

1. The “Research Summaries” page, which contains the PLS of various RECOVER studies and hyperlinks to the externally published full studies.
2. The “Videos” page, which hosts “Discover RECOVER” and other video assets.
3. The “Publications” page, which hosts a separate page for every RECOVER publication, links to their plain language deliverables (summaries and/or videos), and to the externally published article.

Paired t-tests were conducted to determine whether observed differences in website traffic and engagement between the two time periods were statistically significant.

To assess the human oversight required to develop resources with the assistance of generative-AI, we performed a raw count of the number of changes made by human reviewers from the original AI-generated draft to the finalized resource. This assessment was performed on all AI-assisted video scripts and written PLS published between September 10, 2024 and January 20, 2025.

To ensure readability of the AI-enhanced PLS and English-language video scripts, we employed the Flesch-Kincaid Readability Test. This established and widely used metric calculates a grade level equivalent for written text, effectively estimating the years of education required for comprehension.^10^ Flesch-Kincaid Readability levels are a composite of the number of words, sentences, and syllables in a piece of text.^11^ A lower Flesch-Kincaid grade level indicates greater readability and broader accessibility. Given the importance of clear communication in our PLS, we targeted a 6th-grade reading level. While NYUGPT was prompted to generate content at this target level, the initial AI-generated drafts exhibited a significantly higher average reading level of 12th grade. Therefore, the Flesch-Kincaid test served as an objective measure of the discrepancy between the intended and actual complexity of the AI-generated text. Specifically, we assessed the Flesch-Kincaid level for both the initial AI-generated draft and the final, human-revised version of each PLS and English-language video script. This two-stage assessment allowed us to quantify the impact of human editing on improving readability and achieving our target grade level, thereby ensuring the PLS and video scripts are accessible to our audience.

Data collection was performed continuously throughout both time periods, and all data were anonymized and aggregated to maintain privacy and ensure compliance with data protection regulations.

## Results

Between the two time periods, the change in views and active users was not statistically significant. However, there was an average increase in the engagement time of 4.37 seconds (95% CI 0.076s – 8.67s)(*P* = .0461) per active user.

Between the two time periods, there was a general decrease in views and active users on the RECOVER homepage, publications page, and the research summaries page (see Table 1). However, views and active users on the videos page continued to increase between the two time periods, 25% increase in views and 15% increase in active users, suggesting the video content continued to drive interest even as general interest declined.

**Figure 2:**
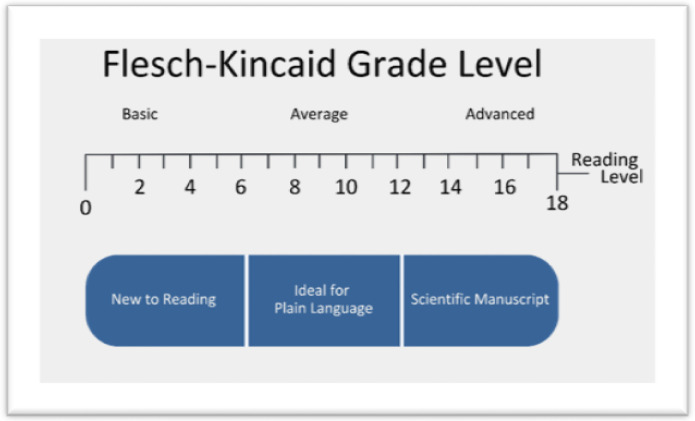
Flesch-Kincaid Grade Level Range The Flesch-Kincaid Grade Level (0–18) assesses text readability, with lower scores indicating easier comprehension. This figure shows the reading level spectrum, from “basic” (0–5) to “advanced” (13–18), with the “average” range (6–12) considered ideal for Plain Language. RECOVER targets a 6th-grade reading level to maximize accessibility for diverse audiences, including patients, caregivers, and the public.

All AI-enhanced deliverables required extensive human revision to ensure that they were clear, concise, accurate, and in plain language. We performed a count of the number of material changes from the initial AI-generated draft to the final product. The mean number of material changes for all AI-enhanced deliverables (n = 17) was 19.875 (SD = 15.046, 95% CI [11.858, 27.892]). We analyzed the Flesch-Kincaid Readability (FKR) grade level, ranging from 0 to 18 (Fig. 2), for the initial AI-generated draft of the content and the final, human-reviewed product. For all AI-enhanced deliverables, the mean FKR level of the initial AI-draft was 12.276 (SD = 1.810, 95% CI [11.346, 13.207]) while the mean FKR level for the final products was 11.56 (SD = 2.129, 95% CI [10.464, 12.654]). While both the original and final products still carry a mean FKR level equivalent to a 12th grade reading level, we observed a 6.02% decrease in the FKR level after human review across all AI-enhanced products (Table 2). This decrease in readability level represents a simplification of the text after human-review.

Figure 2: The Flesch-Kincaid Grade Level (0–18) assesses text readability, with lower scores indicating easier comprehension. This figure shows the reading level spectrum, from “basic” (0–5) to “advanced” (13–18), with the “average” range (6–12) considered ideal for Plain Language. RECOVER targets a 6th-grade reading level to maximize accessibility for diverse audiences, including patients, caregivers, and the public.

Each Publications page is devoted to a different scientific publication, and thus different scientific topics. We used the increase in average user engagement time for each publications page as a surrogate for public interest in various research topics. To determine topics of interest to the end user, we calculated the percent change in average active user engagement time for each publication’s webpage between the two time periods and then evaluated the subject matter of those pages that showed an increase in average engagement time. Based on this analysis, we were able to identify 10 general research topics that continue to garner user engagement (see Table 3). The complete list of topics that saw increased engagement time between the two time periods can be found in S_1: Research topics and subtopics that continue to see increased average user engagement time.

## Discussion

In RECOVER, AI is leveraged to hasten distillation of research for PLS and video presentations. By automating these initial drafts, our teams save nearly 4 hours per project, significantly reducing the typical 10 + hour process. With an anticipated 25–30 manuscripts annually, this translates to a 200–240 hour reduction in workload for PLS development and video scripts. This time saving allows communication teams to dedicate more resources to other projects, e.g. media promotion. This faster turn-around improves web user engagement through timely dissemination of information.

Our analysis revealed the crucial role of human review and quality control in developing effective scientific and health communications. By prioritizing readability, human review can enhance accessibility, facilitating a broader understanding of scientific and health-related information. This improved comprehension is essential for informed decision-making regarding personal health and engagement with scientific advancements. A focus on readability is not merely a stylistic preference, but a critical component of responsible scientific communication and health education.^12^ Our results indicate that AI-generated output often requires substantial editing to meet desired readability targets. Nine out of the twelve AI-generated PLS had a noticeably higher Flesch-Kincaid grade level than the final, human-revised versions. This discrepancy underscores the current limitations of AI in independently producing summaries that consistently adhere to plain language principles. However, with appropriate human oversight, AI tools enable speedier dissemination, improved readability, and greater team efficiency.

By implementing a strategic approach to AI utilization, we aimed to enhance the quality and accessibility of our research summaries and video scripts. This refined approach allowed us to produce summaries and video scripts that were not only accurate but also more engaging and understandable to a broader audience. Subsequently increasing average engagement time, validating the effectiveness of these AI-assisted strategies. Optimizing for readability and relevance, we have successfully captured and maintained public interest in key research topics. This data-driven approach empowers our teams to make informed decisions about future content development, ensuring that our deliverables continue to meet the evolving needs and interests of our audience.

Looking to the future, we would further streamline the communication pipeline between investigators and the public. Information directly from investigators can be challenging to translate, leading to delays and potential misunderstandings. AI has the potential to not only reduce wait times in this process but also expedite the creation of understandable content. Our goal is to achieve consistently simple PLS and video scripts that are highly accessible in multiple languages, ensuring a wider reach.

## Limitations

This study, while demonstrating the potential of AI in generating PLS for scientific research, also highlights important limitations that warrant consideration. A primary limitation is the reliance on a relatively small sample size. Only twelve PLS and five videos (English and Spanish) were generated with AI assistance, limiting the generalizability of our findings. Future research with a larger and more diverse dataset of scientific articles, engagement data, and target reading levels is necessary to validate these initial observations.

While NYUChatGPT shows promise for content generation and translation of technical documents into accessible language, its capabilities may not fully capture the nuance required to communicate complex chronic diseases, particularly as it relates to patients’ lived experiences. Furthermore, it may generate responses with hallucinated research material.^13^ The risk of misrepresenting research findings or presenting them in a distorted fashion trivializing the experiences of marginalized groups remains a significant concern. AI systems may perpetuate erroneous information— amplifying demographic health disparities or supporting since-discredited psychosomatic models of disease, thus preventing patients from getting the help they need.^14^ These concerns are especially relevant for communication aimed at informing decision-making, particularly regarding health outcomes.

Similarly, our framework also has methodological limitations in measuring impact. While viewership metrics can capture surface-level engagements, tracking changes at the level of policy decisions or clinical practice remains significantly more challenging. Our current system effectively measures engagement metrics, such as views, active users, and engagements over time. However, it doesn’t directly measure whether those activities lead to real-world impact, such as improved health or effective policy decisions. As a result, we may overemphasize quantitative metrics at the expense of qualitative real-world impact.

Engagement and web traffic metrics alone cannot provide a complete picture of the effect of AI-generated content on communicating findings. These metrics cannot account for confounding factors such as the general interest in science and Long COVID, the limitations in the granularity of the available data, and the inherent limitations in using Google Analytics data. During our study period, views on the RECOVER homepage dropped 64%, which could indicate a general decreased interest in Long COVID. However, these metrics cannot explain why this decrease occurred. They cannot account for the changing socio-political climate that may impact user engagement. Similarly, we are limited by the granularity of the data available. Our data does not indicate whether a Publication’s summary was interacted with, only that the page was accessed. It is possible that not all users were exposed to AI-generated content when they viewed each page. Our analysis is also subject to the limitations inherent in using Google Analytics data. For example, a user who visits the RECOVER website, clears their browser cookies, and returns to the site, is considered a new user, potentially overestimating the number of unique users. Conversely, if two users visit the RECOVER website on the same device (e.g. a shared computer), they are recorded as a single user, potentially underestimating the number of unique users.

Our work provides empirical evidence of the current capabilities and limitations of AI in a specific context – generating PLS, video scripts, and synthesizing evidence for communications. While AI holds promise, it is not yet a perfect solution and requires careful oversight. The potential for biases in AI algorithms calls for greater transparency in AI-generated content requiring further investigation.^15^ Our findings demonstrate that human input remains essential to refine AI-generated text, ensuring clarity, conciseness, and appropriate readability levels for target audiences. This necessity for human intervention has implications for the efficiency gains promised by AI tools and highlights the importance of incorporating human review into any AI-assisted workflow.

Just as translating science is crucial for informing research, it’s equally relevant to ensure the research reaches the people it affects. Communications teams can leverage AI to synthesize information from diverse sources to develop informed and accurate messaging for various audiences.^16^ Members of these teams are expected to make highly technical research accessible. The use of AI posits itself as a useful tool for making research digestible, however, the quality and reliability of AI-generated summaries depend heavily on the data it’s trained on and the algorithms used.^17^ Once again, human oversight remains critical to ensure accuracy, avoid misinterpretations, and address potential biases that could inadvertently shape communication. The current discourse surrounding AI development reflects a tension between rapid advancement and cautious implementation.^18^

As institutions seek to integrate AI into established practices like scientific and strategic communications, future research should explore the ethical dimensions to ensure responsible and beneficial use of AI in disseminating scientific knowledge and shaping public discourse.^19^ Finally, this study used the Flesch-Kincaid Grade Level as a metric of readability.^20^ Future work should explore other readability metrics and qualitative assessments of PLS and video scripts to provide a more comprehensive evaluation of AI-generated content.^21^

## Conclusion

Integrating AI into scientific communication for the NIH RECOVER program offers opportunities for efficiency in content creation, as demonstrated by NYUChatGPT’s effectiveness in generating PLS without reducing engagement. However, our findings underscore the necessity of human oversight to ensure accuracy, clarity, and accessibility, as AI-generated content often requires revision. While AI modestly increased user engagement, it struggles with the nuances of scientific research and patient experiences, highlighting the risk of misrepresentation in sensitive areas like health disparities. Despite AI’s potential to synthesize and translate information, it carries risks of bias and misinterpretation. Responsible AI implementation in scientific communication demands careful human review, ongoing refinement of AI tools, and a focus on both accuracy and audience sensitivity.

## Supplementary Material

This is a list of supplementary files associated with this preprint. Click to download.

- SupplementaryTable12.docx

## Figures and Tables

**Table 1:** Change in views, active users, and average engagement time

	Views			Active Users			Average engagement time per active user (seconds)		
Page	P1	P2	% Change	P1	P2	% Change	P1	P2	% Change*
**Homepage**	87883	31855	−63.753	56104	20761	−62.996	29.304	22.991	−21.543
**Publications**	17939	8397	−53.191	5179	2604	−49.720	78.215	57.871	−26.010
**Research Summaries**	7891	3317	−57.965	3220	1210	−62.422	74.428	83.799	12.591
**Videos**	536	673	25.560	358	410	14.525	35.824	42.510	18.663
P1 = The time period from December 1, 2023 to September 10, 2024									
P2 = The time period from September 10, 2024 to January 20, 2025									

* Statistically significant, *P* = 0.046.

**Table 2:** Human-reviewer changes and Flesch-Kincaid Grade Levels for all AI-enhanced deliverables

	# of Changes	Final product word count	Al-Draft Grade Level*	Final Draft Grade Level*
**All Al-enhanced deliverables (n = 17)**				
Mean	19.875	283.176	12.276	11.559
SD	15.046	164.101	1.810	2.129
95% Cl	[11.858, 27.892]	[198.803, 367.549]	[11.346, 13.207]	[10.464, 12.654]
**Videos (n = 5)**				
Mean	39.600	502.600	14.040	13.740
SD	7.987	139.581	0.789	1.141
95% Cl	[29.682, 49.518]	[329.288, 675.912]	[13.06, 15.02]	[12.323, 15.157]
**PLS (n = 12)**				
Mean	10.909	191.750	11.542	10.650
SD	5.576	32.886	1.592	1.749
95% Cl	[7.163, 14.655]	[170.855, 212.645]	[10.53, 12.553]	[9.539, 11.761]

* Grade Level = Flesch-Kincaid Readability Grade Level.

**Table 3:** Research topics that continue to see increased average user engagement time

Topic	# of webpages dedicated to this topic with increased average user engagement time
Cardiopulmonary issues	18
Neuropsychiatric issues	17
PASC Characteristics	11
Infectious Disease issues	9
Investigating techniques	8
Miscellaneous	7
Sleep/Fatigue issues	7
Gastrointestinal issues	5
Investigating Lab measures	4
ENT issues	3
OB/GYN issues	2
Genitourinary issues	1

## Data Availability

The data that supports the findings of this study are available in the supplementary material of this article.
